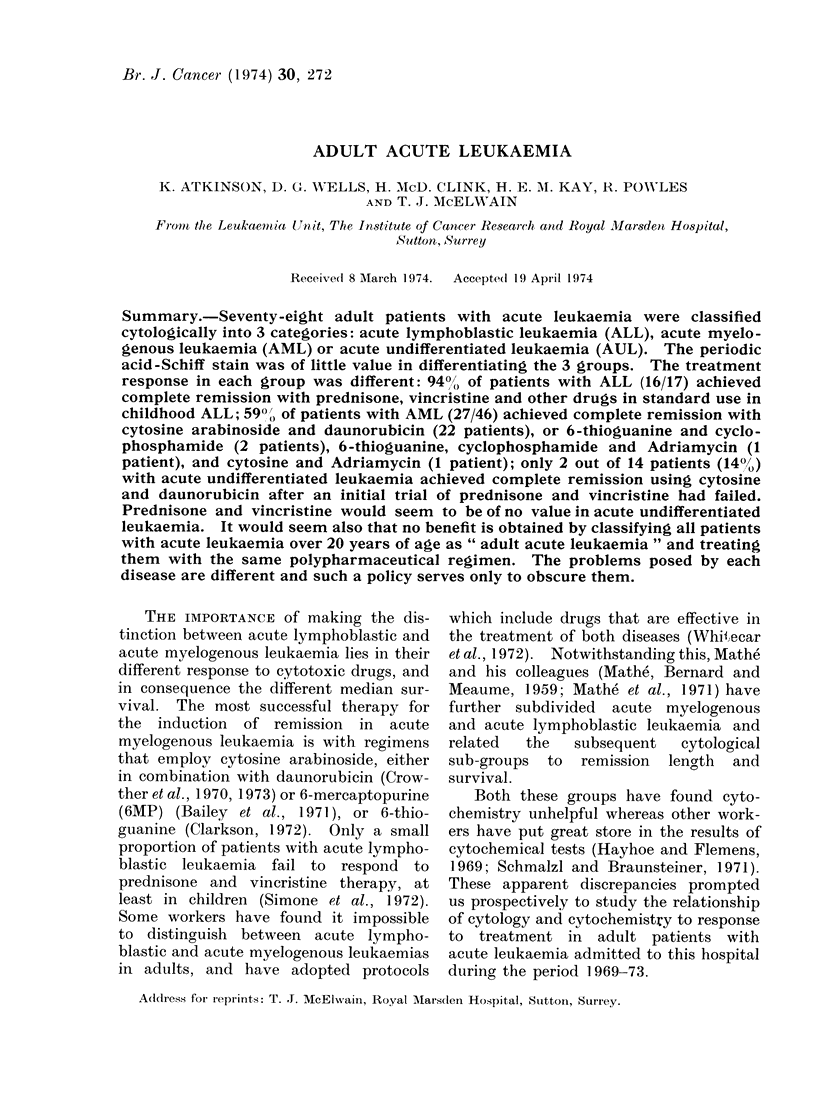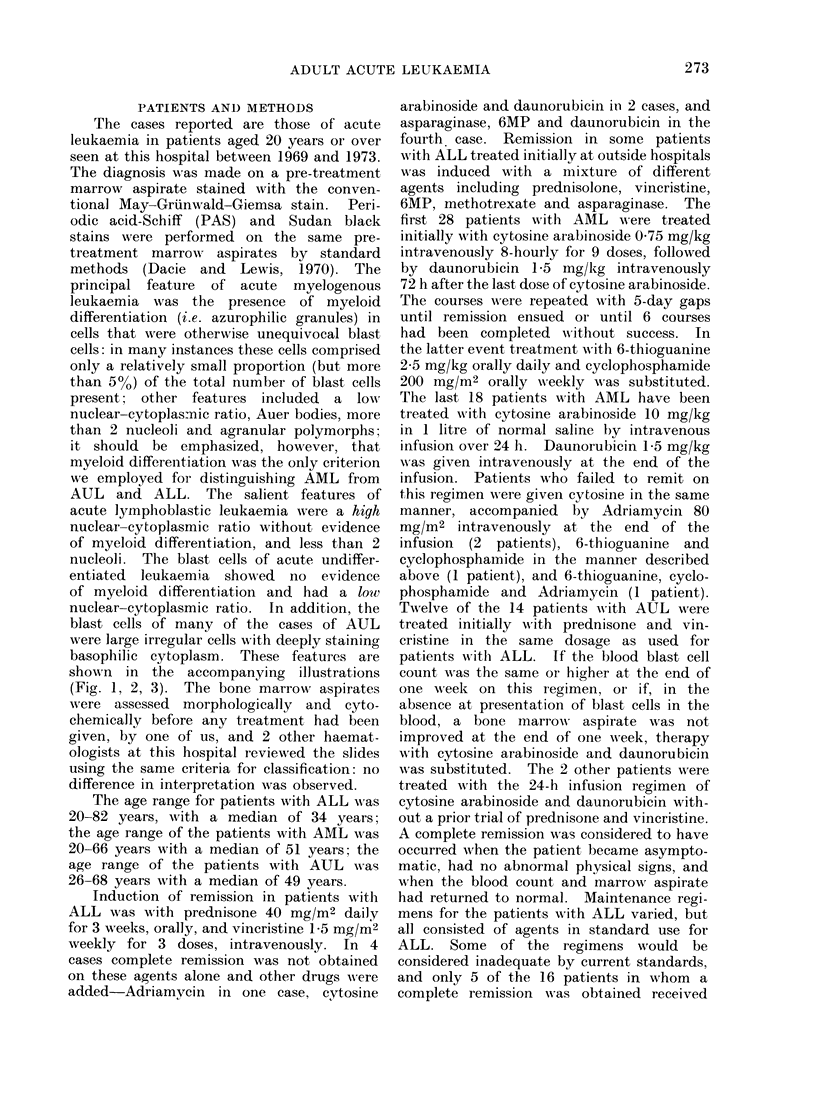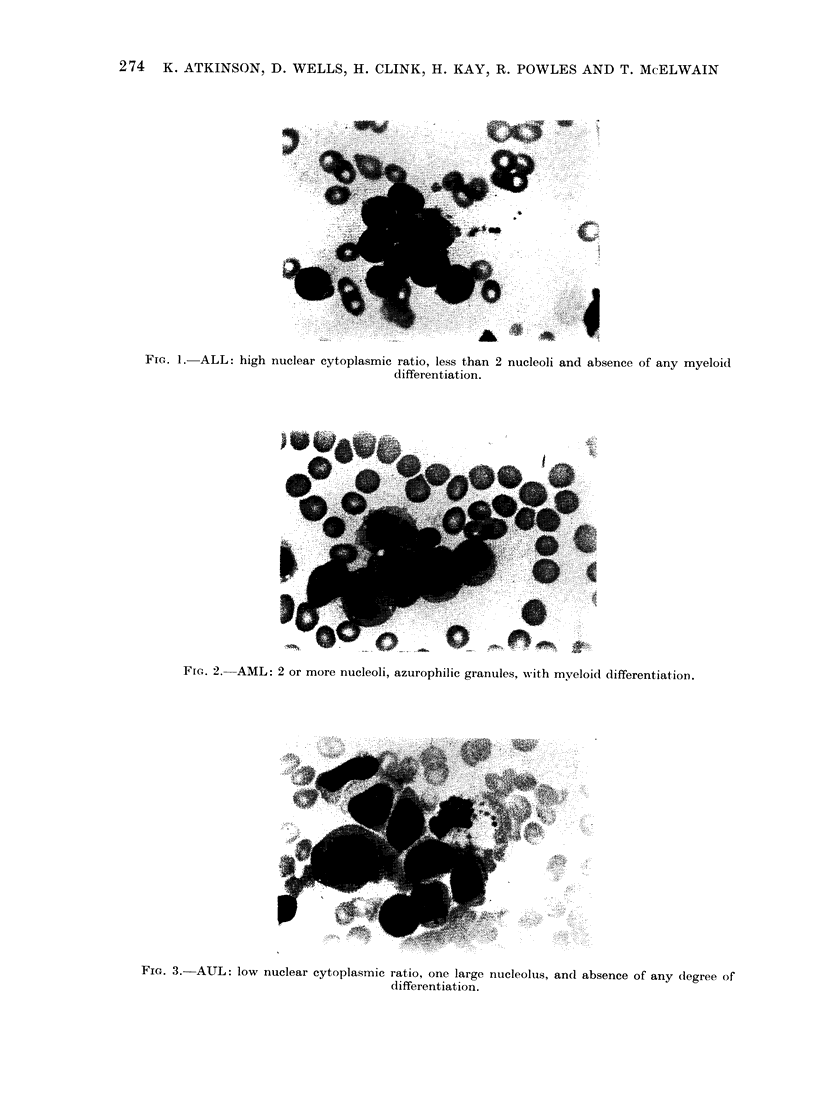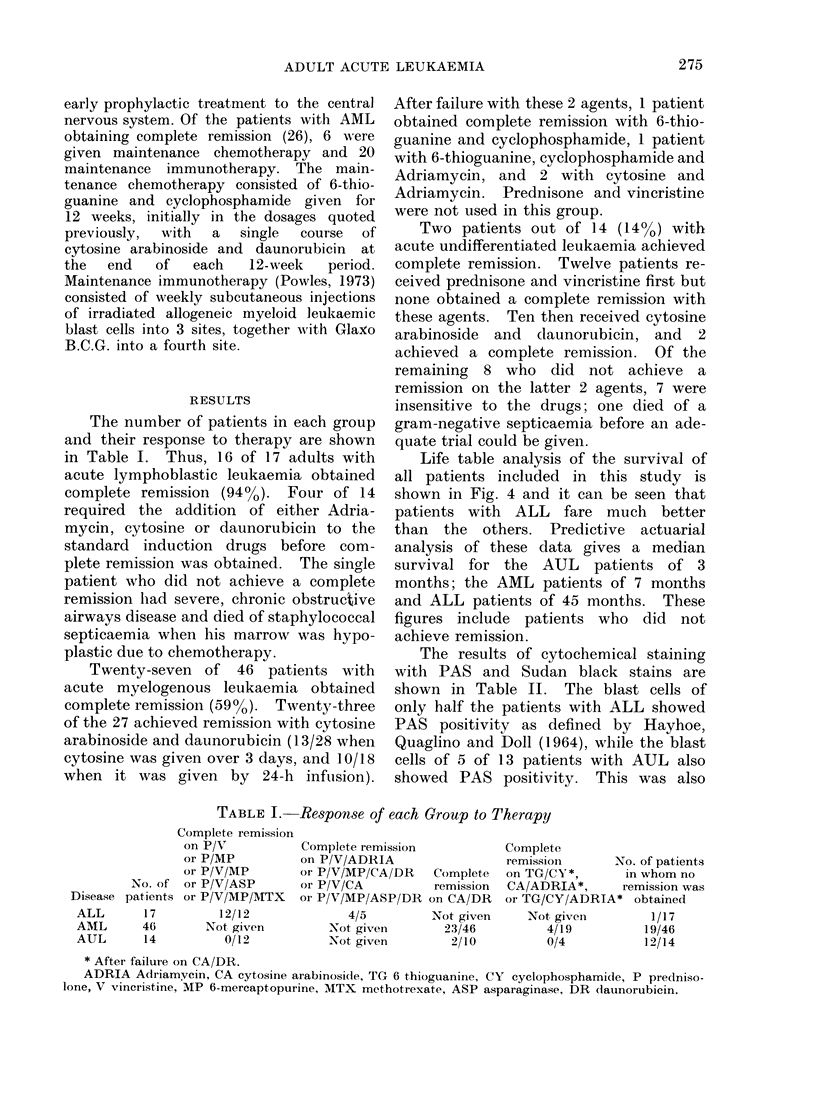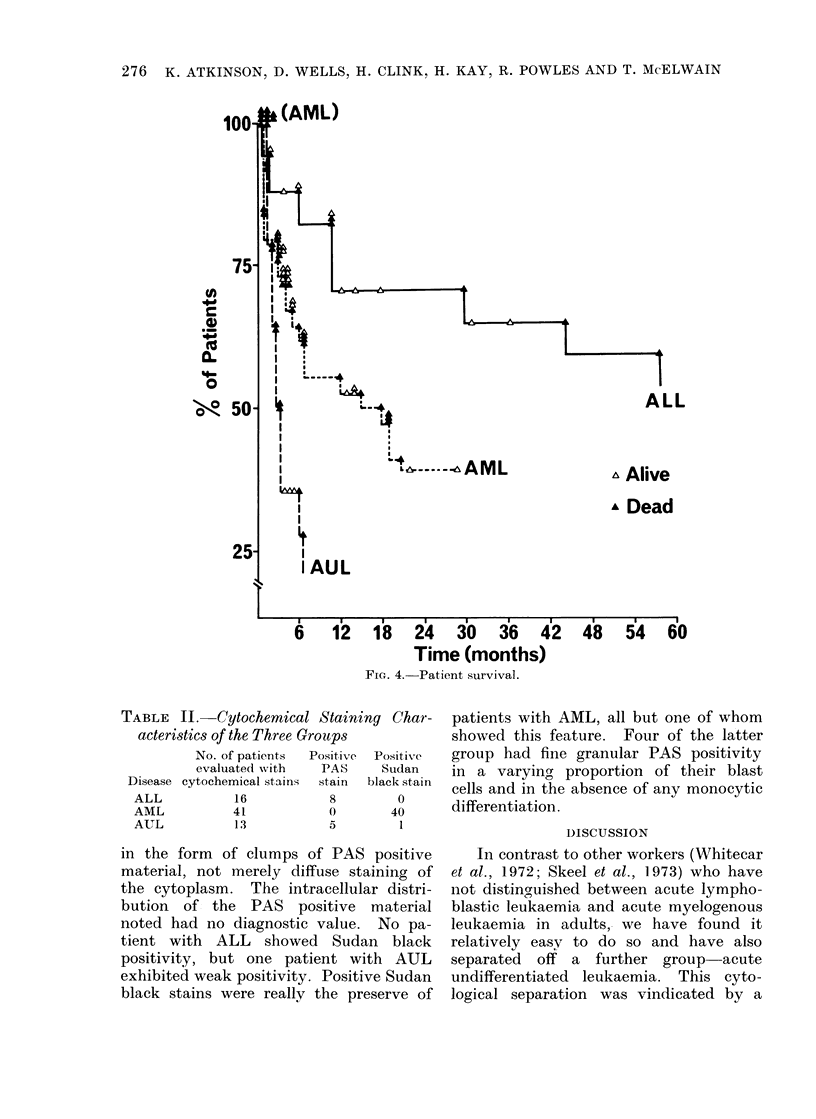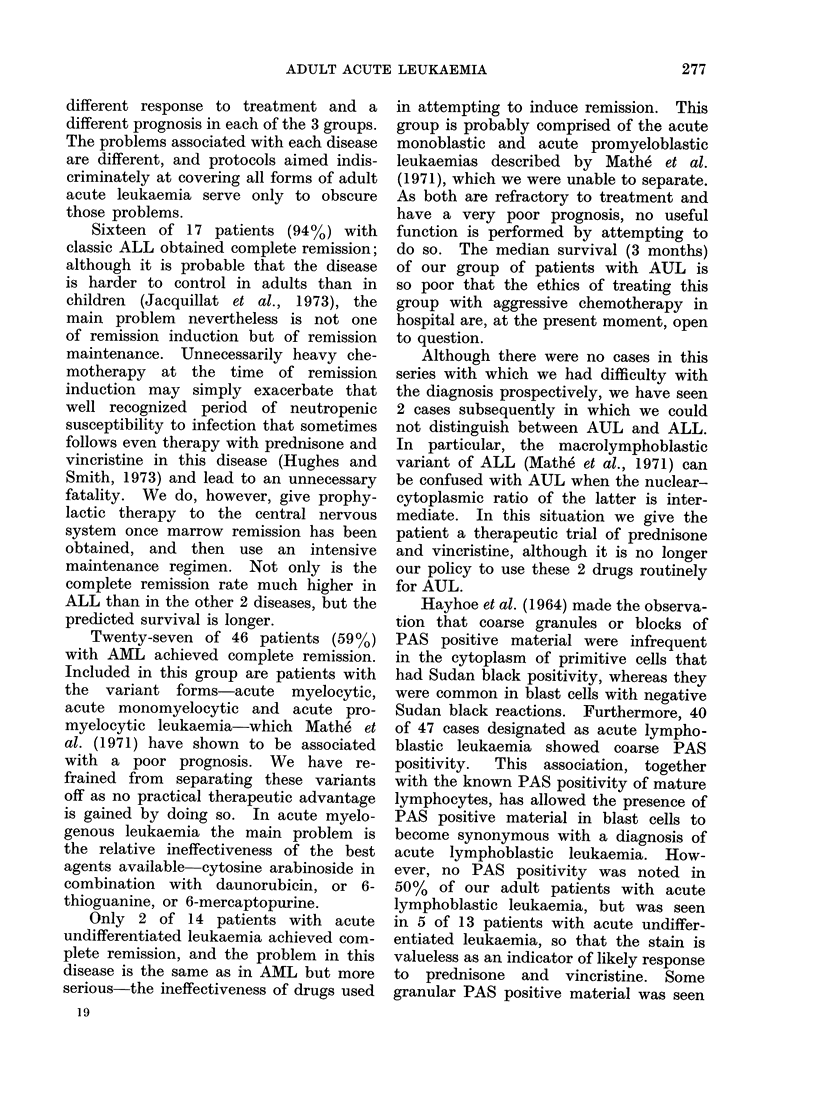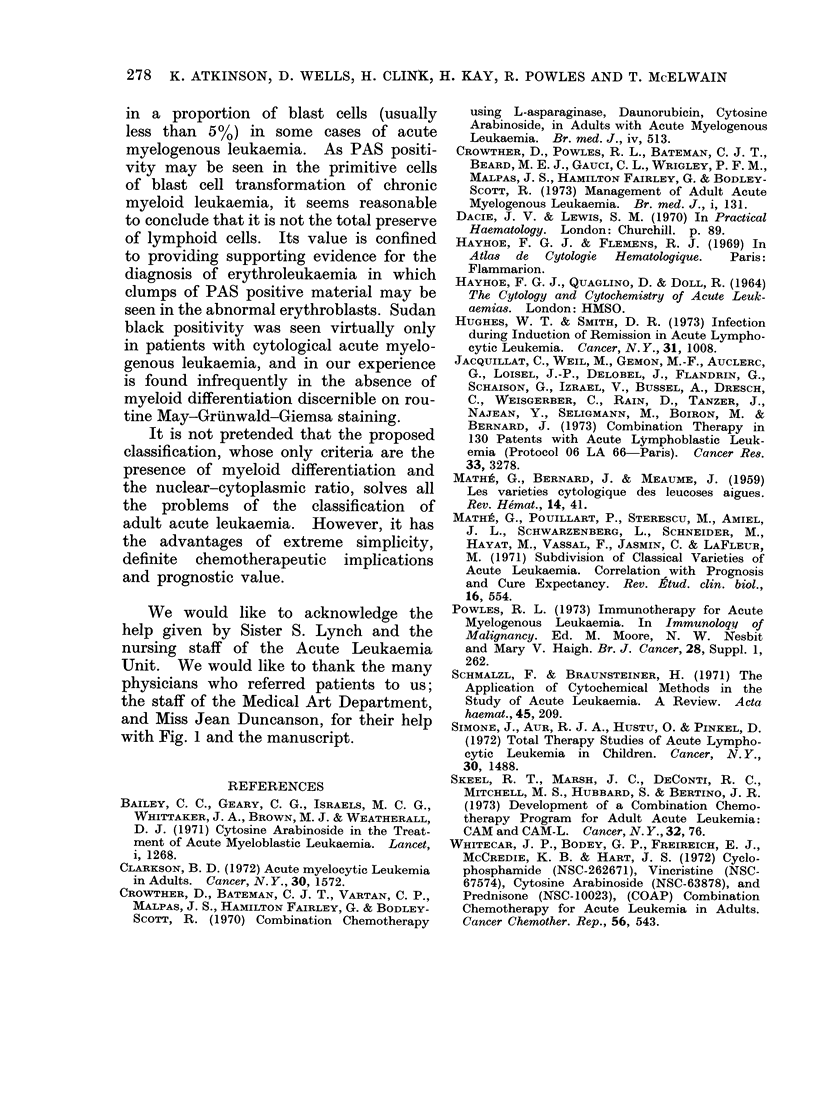# Adult Acute Leukaemia

**DOI:** 10.1038/bjc.1974.191

**Published:** 1974-09

**Authors:** K. Atkinson, D. G. Wells, H. McD. Clink, H. E. M. Kay, R. Powles, T. J. McElwain

## Abstract

**Images:**


Br. J. Cancer (1974) 30, 272

### ADULT ACUTE LEUKAEMIA

K. ATKINSON, D. G. WELLS, H. McD. CLINK, H. E. M. KAY, R. POWLES AND T. J. McELWAIN

*From the Leukaemia Unit, The Institute of Cancer Research and Royal Marsden Hospital, Sutton, Surrey*

Received 8 March 1974. Accepted 19 April 1974

**Summary.—Seventy-eight adult patients with acute leukaemia were classified cytologically into 3 categories: acute lymphoblastic leukaemia (ALL), acute myelogenous leukaemia (AML) or acute undifferentiated leukaemia (AUL). The periodic acid-Schiff stain was of little value in differentiating the 3 groups. The treatment response in each group was different: 94% of patients with ALL (16/17) achieved complete remission with prednisone, vincristine and other drugs in standard use in childhood ALL; 59% of patients with AML (27/46) achieved complete remission with cytosine arabinoside and daunorubicin (22 patients), or 6-thioguanine and cyclophosphamide (2 patients), 6-thioguanine, cyclophosphamide and Adriamycin (1 patient), and cytosine and Adriamycin (1 patient); only 2 out of 14 patients (14%) with acute undifferentiated leukaemia achieved complete remission using cytosine and daunorubicin after an initial trial of prednisone and vincristine had failed. Prednisone and vincristine would seem to be of no value in acute undifferentiated leukaemia. It would seem also that no benefit is obtained by classifying all patients with acute leukaemia over 20 years of age as " adult acute leukaemia " and treating them with the same polypharmaceutical regimen. The problems posed by each disease are different and such a policy serves only to obscure them.**

THE IMPORTANCE of making the distinction between acute lymphoblastic and acute myelogenous leukaemia lies in their different response to cytotoxic drugs, and in consequence the different median survival. The most successful therapy for the induction of remission in acute myelogenous leukaemia is with regimens that employ cytosine arabinoside, either in combination with daunorubicin (Crowther *et al.*, 1970, 1973) or 6-mercaptopurine (6MP) (Bailey *et al.*, 1971), or 6-thioguanine (Clarkson, 1972). Only a small proportion of patients with acute lymphoblastic leukaemia fail to respond to prednisone and vincristine therapy, at least in children (Simone *et al.*, 1972). Some workers have found it impossible to distinguish between acute lymphoblastic and acute myelogenous leukaemias in adults, and have adopted protocols which include drugs that are effective in the treatment of both diseases (Whitecar *et al.*, 1972). Notwithstanding this, Mathé and his colleagues (Mathé, Bernard and Meaume, 1959; Mathé *et al.*, 1971) have further subdivided acute myelogenous and acute lymphoblastic leukaemia and related the subsequent cytological sub-groups to remission length and survival.

Both these groups have found cytochemistry unhelpful whereas other workers have put great store in the results of cytochemical tests (Hayhoe and Flemens, 1969; Schmalzl and Braunsteiner, 1971). These apparent discrepancies prompted us prospectively to study the relationship of cytology and cytochemistry to response to treatment in adult patients with acute leukaemia admitted to this hospital during the period 1969–73.

Address for reprints: T. J. McElwain, Royal Marsden Hospital, Sutton, Surrey.

PATIENTS AND METHODS

The cases reported are those of acute leukaemia in patients aged 20 years or over seen at this hospital between 1969 and 1973. The diagnosis was made on a pre-treatment marrow aspirate stained with the conventional May–Grünwald–Giemsa stain. Periodic acid-Schiff (PAS) and Sudan black stains were performed on the same pre-treatment marrow aspirates by standard methods (Dacie and Lewis, 1970). The principal feature of acute myelogenous leukaemia was the presence of myeloid differentiation (*i.e.* azurophilic granules) in cells that were otherwise unequivocal blast cells: in many instances these cells comprised only a relatively small proportion (but more than 5%) of the total number of blast cells present; other features included a low nuclear–cytoplasmic ratio, Auer bodies, more than 2 nucleoli and agranular polymorphs; it should be emphasized, however, that myeloid differentiation was the only criterion we employed for distinguishing AML from AUL and ALL. The salient features of acute lymphoblastic leukaemia were a *high* nuclear–cytoplasmic ratio without evidence of myeloid differentiation, and less than 2 nucleoli. The blast cells of acute undifferentiated leukaemia showed no evidence of myeloid differentiation and had a *low* nuclear–cytoplasmic ratio. In addition, the blast cells of many of the cases of AUL were large irregular cells with deeply staining basophilic cytoplasm. These features are shown in the accompanying illustrations (Fig. 1, 2, 3). The bone marrow aspirates were assessed morphologically and cytochemically before any treatment had been given, by one of us, and 2 other haematologists at this hospital reviewed the slides using the same criteria for classification: no difference in interpretation was observed.

The age range for patients with ALL was 20–82 years, with a median of 34 years; the age range of the patients with AML was 20–66 years with a median of 51 years; the age range of the patients with AUL was 26–68 years with a median of 49 years.

Induction of remission in patients with ALL was with prednisone 40 mg/m$^2$ daily for 3 weeks, orally, and vincristine 1·5 mg/m$^2$ weekly for 3 doses, intravenously. In 4 cases complete remission was not obtained on these agents alone and other drugs were added—Adriamycin in one case, cytosine arabinoside and daunorubicin in 2 cases, and asparaginase, 6MP and daunorubicin in the fourth case. Remission in some patients with ALL treated initially at outside hospitals was induced with a mixture of different agents including prednisolone, vincristine, 6MP, methotrexate and asparaginase. The first 28 patients with AML were treated initially with cytosine arabinoside 0·75 mg/kg intravenously 8-hourly for 9 doses, followed by daunorubicin 1·5 mg/kg intravenously 72 h after the last dose of cytosine arabinoside. The courses were repeated with 5-day gaps until remission ensued or until 6 courses had been completed without success. In the latter event treatment with 6-thioguanine 2·5 mg/kg orally daily and cyclophosphamide 200 mg/m$^2$ orally weekly was substituted. The last 18 patients with AML have been treated with cytosine arabinoside 10 mg/kg in 1 litre of normal saline by intravenous infusion over 24 h. Daunorubicin 1·5 mg/kg was given intravenously at the end of the infusion. Patients who failed to remit on this regimen were given cytosine in the same manner, accompanied by Adriamycin 80 mg/m$^2$ intravenously at the end of the infusion (2 patients), 6-thioguanine and cyclophosphamide in the manner described above (1 patient), and 6-thioguanine, cyclophosphamide and Adriamycin (1 patient). Twelve of the 14 patients with AUL were treated initially with prednisone and vincristine in the same dosage as used for patients with ALL. If the blood blast cell count was the same or higher at the end of one week on this regimen, or if, in the absence at presentation of blast cells in the blood, a bone marrow aspirate was not improved at the end of one week, therapy with cytosine arabinoside and daunorubicin was substituted. The 2 other patients were treated with the 24-h infusion regimen of cytosine arabinoside and daunorubicin without a prior trial of prednisone and vincristine. A complete remission was considered to have occurred when the patient became asymptomatic, had no abnormal physical signs, and when the blood count and marrow aspirate had returned to normal. Maintenance regimens for the patients with ALL varied, but all consisted of agents in standard use for ALL. Some of the regimens would be considered inadequate by current standards, and only 5 of the 16 patients in whom a complete remission was obtained received

FIG. 1.—ALL: high nuclear cytoplasmic ratio, less than 2 nucleoli and absence of any myeloid differentiation.

FIG. 2.—AML: 2 or more nucleoli, azurophilic granules, with myeloid differentiation.

FIG. 3.—AUL: low nuclear cytoplasmic ratio, one large nucleolus, and absence of any degree of differentiation.

early prophylactic treatment to the central nervous system. Of the patients with AML obtaining complete remission (26), 6 were given maintenance chemotherapy and 20 maintenance immunotherapy. The maintenance chemotherapy consisted of 6-thioguanine and cyclophosphamide given for 12 weeks, initially in the dosages quoted previously, with a single course of cytosine arabinoside and daunorubicin at the end of each 12-week period. Maintenance immunotherapy (Powles, 1973) consisted of weekly subcutaneous injections of irradiated allogeneic myeloid leukaemic blast cells into 3 sites, together with Glaxo B.C.G. into a fourth site.

### RESULTS

The number of patients in each group and their response to therapy are shown in Table I. Thus, 16 of 17 adults with acute lymphoblastic leukaemia obtained complete remission (94%). Four of 14 required the addition of either Adriamycin, cytosine or daunorubicin to the standard induction drugs before complete remission was obtained. The single patient who did not achieve a complete remission had severe, chronic obstructive airways disease and died of staphylococcal septicaemia when his marrow was hypoplastic due to chemotherapy.

Twenty-seven of 46 patients with acute myelogenous leukaemia obtained complete remission (59%). Twenty-three of the 27 achieved remission with cytosine arabinoside and daunorubicin (13/28 when cytosine was given over 3 days, and 10/18 when it was given by 24-h infusion). After failure with these 2 agents, 1 patient obtained complete remission with 6-thioguanine and cyclophosphamide, 1 patient with 6-thioguanine, cyclophosphamide and Adriamycin, and 2 with cytosine and Adriamycin. Prednisone and vincristine were not used in this group.

Two patients out of 14 (14%) with acute undifferentiated leukaemia achieved complete remission. Twelve patients received prednisone and vincristine first but none obtained a complete remission with these agents. Ten then received cytosine arabinoside and daunorubicin, and 2 achieved a complete remission. Of the remaining 8 who did not achieve a remission on the latter 2 agents, 7 were insensitive to the drugs; one died of a gram-negative septicaemia before an adequate trial could be given.

Life table analysis of the survival of all patients included in this study is shown in Fig. 4 and it can be seen that patients with ALL fare much better than the others. Predictive actuarial analysis of these data gives a median survival for the AUL patients of 3 months; the AML patients of 7 months and ALL patients of 45 months. These figures include patients who did not achieve remission.

The results of cytochemical staining with PAS and Sudan black stains are shown in Table II. The blast cells of only half the patients with ALL showed PAS positivity as defined by Hayhoe, Quaglino and Doll (1964), while the blast cells of 5 of 13 patients with AUL also showed PAS positivity. This was also

TABLE I.—*Response of each Group to Therapy*

| Disease | No. of patients | Complete remission on P/V or P/MP or P/V/MP or P/V/ASP or P/V/MP/MTX | Complete remission on P/V/ADRIA or P/V/MP/CA/DR or P/V/CA or P/V/MP/ASP/DR | Complete remission on CA/DR | Complete remission on TG/CY*, CA/ADRIA*, or TG/CY/ADRIA* | No. of patients in whom no remission was obtained |
|---|---|---|---|---|---|---|
| ALL | 17 | 12/12 | 4/5 | Not given | Not given | 1/17 |
| AML | 46 | Not given | Not given | 23/46 | 4/19 | 19/46 |
| AUL | 14 | 0/12 | Not given | 2/10 | 0/4 | 12/14 |

* After failure on CA/DR.

ADRIA Adriamycin, CA cytosine arabinoside, TG 6 thioguanine, CY cyclophosphamide, P prednisolone, V vincristine, MP 6-mercaptopurine, MTX methotrexate, ASP asparaginase, DR daunorubicin.

FIG. 4.—Patient survival.

TABLE II.—*Cytochemical Staining Characteristics of the Three Groups*

| Disease | No. of patients evaluated with cytochemical stains | Positive PAS stain | Positive Sudan black stain |
|---|---|---|---|
| ALL | 16 | 8 | 0 |
| AML | 41 | 0 | 40 |
| AUL | 13 | 5 | 1 |

in the form of clumps of PAS positive material, not merely diffuse staining of the cytoplasm. The intracellular distribution of the PAS positive material noted had no diagnostic value. No patient with ALL showed Sudan black positivity, but one patient with AUL exhibited weak positivity. Positive Sudan black stains were really the preserve of patients with AML, all but one of whom showed this feature. Four of the latter group had fine granular PAS positivity in a varying proportion of their blast cells and in the absence of any monocytic differentiation.

### DISCUSSION

In contrast to other workers (Whitecar *et al.*, 1972; Skeel *et al.*, 1973) who have not distinguished between acute lymphoblastic leukaemia and acute myelogenous leukaemia in adults, we have found it relatively easy to do so and have also separated off a further group—acute undifferentiated leukaemia. This cytological separation was vindicated by a different response to treatment and a different prognosis in each of the 3 groups. The problems associated with each disease are different, and protocols aimed indiscriminately at covering all forms of adult acute leukaemia serve only to obscure those problems.

Sixteen of 17 patients (94%) with classic ALL obtained complete remission; although it is probable that the disease is harder to control in adults than in children (Jacquillat *et al.*, 1973), the main problem nevertheless is not one of remission induction but of remission maintenance. Unnecessarily heavy chemotherapy at the time of remission induction may simply exacerbate that well recognized period of neutropenic susceptibility to infection that sometimes follows even therapy with prednisone and vincristine in this disease (Hughes and Smith, 1973) and lead to an unnecessary fatality. We do, however, give prophylactic therapy to the central nervous system once marrow remission has been obtained, and then use an intensive maintenance regimen. Not only is the complete remission rate much higher in ALL than in the other 2 diseases, but the predicted survival is longer.

Twenty-seven of 46 patients (59%) with AML achieved complete remission. Included in this group are patients with the variant forms—acute myelocytic, acute monomyelocytic and acute promyelocytic leukaemia—which Mathé *et al.* (1971) have shown to be associated with a poor prognosis. We have refrained from separating these variants off as no practical therapeutic advantage is gained by doing so. In acute myelogenous leukaemia the main problem is the relative ineffectiveness of the best agents available—cytosine arabinoside in combination with daunorubicin, or 6-thioguanine, or 6-mercaptopurine.

Only 2 of 14 patients with acute undifferentiated leukaemia achieved complete remission, and the problem in this disease is the same as in AML but more serious—the ineffectiveness of drugs used in attempting to induce remission. This group is probably comprised of the acute monoblastic and acute promyeloblastic leukaemias described by Mathé *et al.* (1971), which we were unable to separate. As both are refractory to treatment and have a very poor prognosis, no useful function is performed by attempting to do so. The median survival (3 months) of our group of patients with AUL is so poor that the ethics of treating this group with aggressive chemotherapy in hospital are, at the present moment, open to question.

Although there were no cases in this series with which we had difficulty with the diagnosis prospectively, we have seen 2 cases subsequently in which we could not distinguish between AUL and ALL. In particular, the macrolymphoblastic variant of ALL (Mathé *et al.*, 1971) can be confused with AUL when the nuclear–cytoplasmic ratio of the latter is intermediate. In this situation we give the patient a therapeutic trial of prednisone and vincristine, although it is no longer our policy to use these 2 drugs routinely for AUL.

Hayhoe *et al.* (1964) made the observation that coarse granules or blocks of PAS positive material were infrequent in the cytoplasm of primitive cells that had Sudan black positivity, whereas they were common in blast cells with negative Sudan black reactions. Furthermore, 40 of 47 cases designated as acute lymphoblastic leukaemia showed coarse PAS positivity. This association, together with the known PAS positivity of mature lymphocytes, has allowed the presence of PAS positive material in blast cells to become synonymous with a diagnosis of acute lymphoblastic leukaemia. However, no PAS positivity was noted in 50% of our adult patients with acute lymphoblastic leukaemia, but was seen in 5 of 13 patients with acute undifferentiated leukaemia, so that the stain is valueless as an indicator of likely response to prednisone and vincristine. Some granular PAS positive material was seen in a proportion of blast cells (usually less than 5%) in some cases of acute myelogenous leukaemia. As PAS positivity may be seen in the primitive cells of blast cell transformation of chronic myeloid leukaemia, it seems reasonable to conclude that it is not the total preserve of lymphoid cells. Its value is confined to providing supporting evidence for the diagnosis of erythroleukaemia in which clumps of PAS positive material may be seen in the abnormal erythroblasts. Sudan black positivity was seen virtually only in patients with cytological acute myelogenous leukaemia, and in our experience is found infrequently in the absence of myeloid differentiation discernible on routine May–Grünwald–Giemsa staining.

It is not pretended that the proposed classification, whose only criteria are the presence of myeloid differentiation and the nuclear–cytoplasmic ratio, solves all the problems of the classification of adult acute leukaemia. However, it has the advantages of extreme simplicity, definite chemotherapeutic implications and prognostic value.

We would like to acknowledge the help given by Sister S. Lynch and the nursing staff of the Acute Leukaemia Unit. We would like to thank the many physicians who referred patients to us; the staff of the Medical Art Department, and Miss Jean Duncanson, for their help with Fig. 1 and the manuscript.

### REFERENCES

Bailey, C. C., Geary, C. G., Israels, M. C. G., Whittaker, J. A., Brown, M. J. & Weatherall, D. J. (1971) Cytosine Arabinoside in the Treatment of Acute Myeloblastic Leukaemia. *Lancet*, i, 1268.

Clarkson, B. D. (1972) Acute myelocytic Leukemia in Adults. *Cancer, N.Y.*, **30**, 1572.

Crowther, D., Bateman, C. J. T., Vartan, C. P., Malpas, J. S., Hamilton Fairley, G. & Bodley-Scott, R. (1970) Combination Chemotherapy using L-asparaginase, Daunorubicin, Cytosine Arabinoside, in Adults with Acute Myelogenous Leukaemia. *Br. med. J.*, iv, 513.

Crowther, D., Powles, R. L., Bateman, C. J. T., Beard, M. E. J., Gauci, C. L., Wrigley, P. F. M., Malpas, J. S., Hamilton Fairley, G. & Bodley-Scott, R. (1973) Management of Adult Acute Myelogenous Leukaemia. *Br. med. J.*, i, 131.

Dacie, J. V. & Lewis, S. M. (1970) In *Practical Haematology*. London: Churchill. p. 89.

Hayhoe, F. G. J. & Flemens, R. J. (1969) In *Atlas de Cytologie Hematologique*. Paris: Flammarion.

Hayhoe, F. G. J., Quaglino, D. & Doll, R. (1964) *The Cytology and Cytochemistry of Acute Leukaemias*. London: HMSO.

Hughes, W. T. & Smith, D. R. (1973) Infection during Induction of Remission in Acute Lymphocytic Leukemia. *Cancer, N.Y.*, **31**, 1008.

Jacquillat, C., Weil, M., Gemon, M.-F., Auclerc, G., Loisel, J.-P., Delobel, J., Flandrin, G., Schaison, G., Izrael, V., Bussel, A., Dresch, C., Weisgerber, C., Rain, D., Tanzer, J., Najean, Y., Seligmann, M., Boiron, M. & Bernard, J. (1973) Combination Therapy in 130 Patents with Acute Lymphoblastic Leukemia (Protocol 06 LA 66—Paris). *Cancer Res.* **33**, 3278.

Mathé, G., Bernard, J. & Meaume, J. (1959) Les varieties cytologique des leucoses aigues. *Rev. Hémat.*, **14**, 41.

Mathé, G., Pouillart, P., Sterescu, M., Amiel, J. L., Schwarzenberg, L., Schneider, M., Hayat, M., Vassal, F., Jasmin, C. & LaFleur, M. (1971) Subdivision of Classical Varieties of Acute Leukaemia. Correlation with Prognosis and Cure Expectancy. *Rev. Étud. clin. biol.*, **16**, 554.

Powles, R. L. (1973) Immunotherapy for Acute Myelogenous Leukaemia. In *Immunoloqy of Malignancy*. Ed. M. Moore, N. W. Nesbit and Mary V. Haigh. *Br. J. Cancer*, **28**, Suppl. 1, 262.

Schmalzl, F. & Braunsteiner, H. (1971) The Application of Cytochemical Methods in the Study of Acute Leukaemia. A Review. *Acta haemat.*, **45**, 209.

Simone, J., Aur, R. J. A., Hustu, O. & Pinkel, D. (1972) Total Therapy Studies of Acute Lymphocytic Leukemia in Children. *Cancer, N.Y.*, **30**, 1488.

Skeel, R. T., Marsh, J. C., DeConti, R. C., Mitchell, M. S., Hubbard, S. & Bertino, J. R. (1973) Development of a Combination Chemotherapy Program for Adult Acute Leukemia: CAM and CAM-L. *Cancer, N.Y.*, **32**, 76.

Whitecar, J. P., Bodey, G. P., Freireich, E. J., McCredie, K. B. & Hart, J. S. (1972) Cyclophosphamide (NSC-262671), Vincristine (NSC-67574), Cytosine Arabinoside (NSC-63878), and Prednisone (NSC-10023), (COAP) Combination Chemotherapy for Acute Leukemia in Adults. *Cancer Chemother. Rep.*, **56**, 543.